# The Role of Keratin17 in Human Tumours

**DOI:** 10.3389/fcell.2022.818416

**Published:** 2022-02-24

**Authors:** Hanqun Zhang, Yun Zhang, Tingting Xia, Liang Lu, Min Luo, Yanping Chen, Yuncong Liu, Yong Li

**Affiliations:** ^1^ Department of Oncology, Guizhou Provincial People’s Hospital, Guizhou, China; ^2^ Department of Pathology, Guizhou Provincial People’s Hospital, Guizhou, China; ^3^ Department of Nephrology, Guizhou Provincial People’s Hospital, Guizhou, China

**Keywords:** Krt17, biomarker, prognostic, malignant, and cancer

## Abstract

Keratins are a group of proteins that can constitute intermediate fibers. It is a component of the cytoskeleton and plays an important role in cell protection and structural support. Keratin 17, a Type I keratin, is a multifunctional protein that regulates a variety of biological processes, including cell growth, proliferation, migration, apoptosis and signal transduction. Abnormal expression of KRT17 is associated with a variety of diseases, such as skin diseases. In recent years, studies have shown that KRT17 is abnormally expressed in a variety of malignant tumours, such as lung cancer, cervical cancer, oral squamous cell carcinoma and sarcoma. These abnormal expressions are related to the occurrence, development and prognosis of malignant tumors. In this review, we summarized the expression patterns of KRT17 in a variety of malignant tumours, the role of KRT17 in the development and prognosis of different malignant tumors and its molecular mechanisms. We also discuss the potential clinical application of KRT17 as a valuable therapeutic target.

## Introduction

Malignant tumours are considered a serious threat and one of the leading causes of death worldwide. In addition, with the increase of the global population and unhealthy lifestyles, the situation will become more serious (Torre et al., 2016). Although surgery, radiotherapy, chemotherapy and immunotherapy have been great progress, malignant tumours remain a serious challenge to the clinicians and the researchers worldwide (Zugazagoitia et al., 2016; Cutsem et al., 2016; Hegde and Chen, 2020). One of the most important factors for the poor prognosis of malignant tumours is the lack of effective pre-progressive diagnosis methods and effective prognostic indicators to guide clinical diagnosis and treatment. Therefore, it is important to find suitable molecular biomarkers for the diagnosis of malignant tumours to guide clinical decision-making (Yang et al., 2020).

Keratin is an important part of the cytoskeleton and a member of the intermediate filament superfamily. The intermediate filament (IF) cytoskeleton of mammalian epithelia is generated from pairs of type I and type II keratins, which are encoded by two large gene families and consist of 54 genes in humans and the mice (Vijayaraj et al., 2007). According to gene substructure and nucleotide sequence homology, keratin can be divided into two types: 28 Type I acidic proteins and 26 Type II basic proteins. The closure of embryonic wounds is significantly delayed in KRT17 null embryos (Mazzalupo et al., 2003). KRT17 binds to 14-3-3σ to regulate protein synthesis to influence cell growth and keratin size in mice (Kim et al., 2006). In addition, KRT17 knockout increased hair fragility and hair stromal cell apoptosis, resulting in transient hair loss after birth in mice (McGowan et al., 2002). In human disease, Keratin plays an important role in maintaining cell integrity, regulating cell growth and migration and protecting cells from apoptosis (Jacob et al., 2018).

**FIGURE 1:**
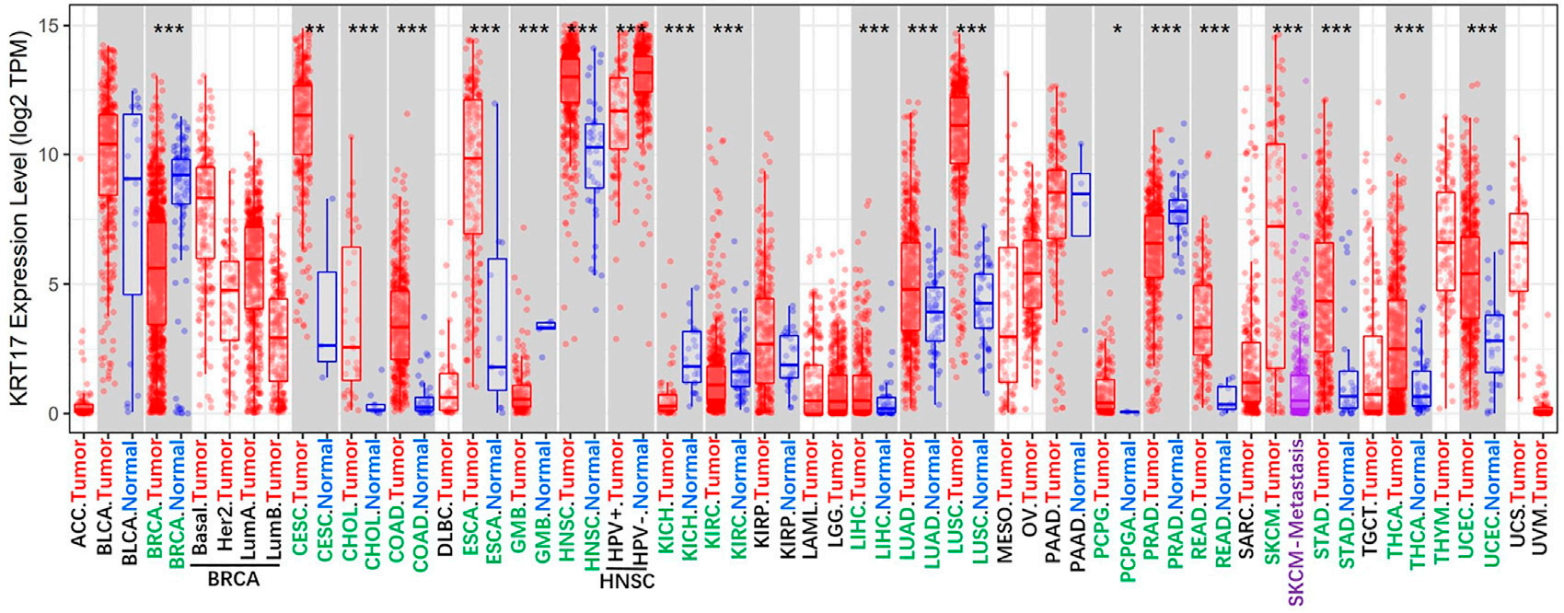
The expression status of the KRT17 in 33 kinds of malignant tumour tissues and 22 kinds of normal tissues. (TCGA dataset) (http://timer.cistrome.org/) (*p < 0.05; **p < 0 01; ***p < 0 001).

KRT17 is a type I keratin that is commonly expressed in epithelial cells (Kurokawa et al., 2011; Yang D et al., 2019). KRT17 is not expressed in normal skin, but its expression can be induced under stress conditions, such as skin scratching (McGowan and Coulombe, 1998). In recent years, abnormal expression of KRT17 has been found in malignant tumours, and these abnormal expressions were the mainly high expression (Figure 1), and this high expression is related to the occurrence, development and prognosis of malignant tumours. In this review, we summarise the current evidence on the role of KRT17 in malignant tumours, including those focused on its abnormal expression, biological functions, molecular mechanisms and associated clinical features. Our goal is to provide useful information for clinical research and application of KRT17.

## Structure and Function of Keratin 17

**FIGURE 2:**
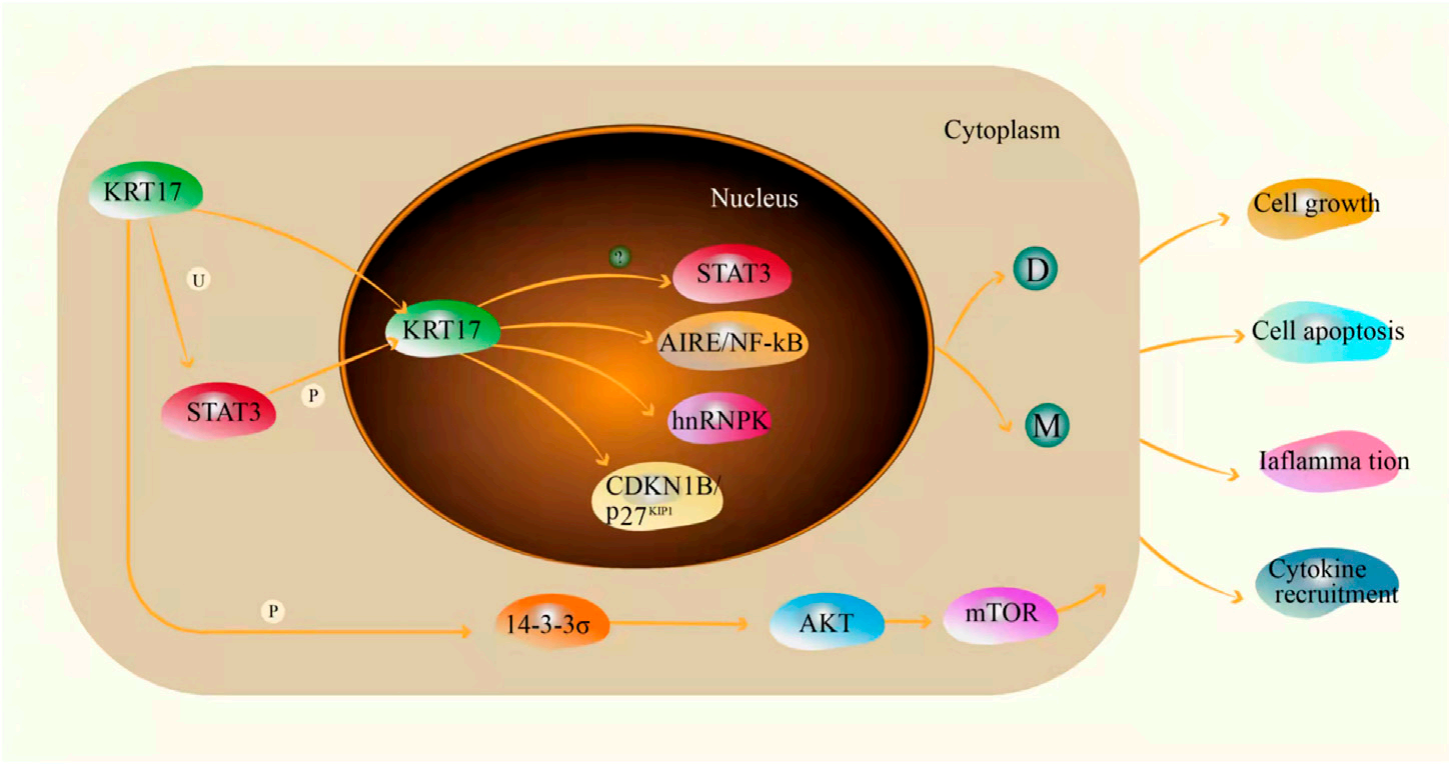
The mechanism of KRT17 in cells(U: ubiquitin; P: phosphorylated; D: degradation; M: mature fully polymerized keratin filaments; ?: unknown).

Keratin 17 (KRT17) belongs to the family of Type I intermediate keratins. Intermediate filaments (IFs) are a new cytoskeletal filament assembly between actin microfilaments (∼6–8 nm) and microtubules (∼25 nm). The diameter is ∼10 nm (Ishikawa et al., 1968). Like all members of the keratin family, KRT17 is a triplet structure consisting of 432 amino acids. It has a non-helical head (1–83), an α-helix (84–392) and a non-helical tail domain (393–432) (Yang L et al., 2019). The KRT17 gene is located on chromosome 17q21.2 (Kurokawa et al., 2011). KRT17 is a multifunctional protein that regulates a variety of cellular processes, including cell proliferation, growth and apoptosis (Depianto et al., 2010; Mikami et al., 2015; Zeng et al., 2020). KRT17 is one of the components of intermediate filaments and mainly plays a role in the cytoplasm. In the cytoplasm, when KRT17 is ubiquitinated, it can interact with STAT3 to promote cell proliferation (Yang et al., 2018), but this effect may still exist in the nucleus. When phosphorylation of KRT17 increases the level of 14-3-3σ in the cytoplasm, phosphorylated KRT17 binds to 14-3-3σ and promotes cell growth through the AKT/mTOR pathway. When phosphorylation of KRT17 is low, 14-3-3σ enters the nucleus and inhibits cell growth and protein synthesis (Kim et al., 2006). In addition, KRT17 can also promote the release of inflammatory cytokines, and promote the occurrence and development of skin tumours by inducing inflammation (Chung et al., 2015; Lo et al., 2010). It may be associated with skin tumours or played a role in the development of skin tumours. The abnormal expression of KRT17 was initially identified as the dominant cytokeratin in basal cell carcinoma of the skin (Moll et al., 1982). However, recent studies have shown that KRT17 can shuttle between inside and outside the nucleus due to the presence of nuclear localization signals and nuclear output signals. This new idea of nuclear localization of intermediate filaments raises the possibility that keratin regulates additional cellular processes (Hobbs et al., 2016). Studies have shown that Autoimmune regulator (Aire), a transcriptional regulator, induces KRT17 dependent expression in human and mouse tumour keratinocytes and requires GLI-2 induced skin tumour development in mice. Induction of Aire mRNA in keratinocytes depends on the functional interaction between KRT17 and the heterogeneous nuclear ribonucleoprotein hnRNP K. Furthermore, KRT17 co-locates with Aire proteins in the nucleus of tumour keratinocytes, and each binds to specific promoter regions characterized by a common sequence of NF-κB in related subsets of KRT17 and Aire dependent pro-inflammatory genes. These results suggest that KRT17 is involved in the process of acute inflammation and tumorigenesis, and provides a molecular basis for KRT17 to promote inflammation and tumorigenesis (Hobbs et al., 2015). In addition, KRT17 regulates cell transition from G1 phase to S phase by promoting the nuclear output and inactivation of CDKN1B/p27KIP1, leading to degradation or maturation of keratin filaments to affect cell function (Escobar-Hoyos et al., 2015) (Figure 2). In recent years, studies have shown that KRT17 is abnormally expressed in a variety of malignant tumours, and up-regulated in lung cancer, pancreatic cancer, gastric cancer and sarcoma (Liu et al., 2018; Chen et al., 2020; Chivu-Economescu et al., 2017; Yan et al., 2020). In addition, Among KRT17 mutations, c.275A > G missense mutations that cause asparagine to be replaced by serine (Asn92Ser) are the most common (Ofaiche et al., 2014; Cogulu et al., 2009). The change at this point may be the main reason for the occurrence and development of malignant tumours caused by KRT17, but the specific molecular mechanism is still not clear, and needs further study. Furthermore, the high expression of KRT17 can promote tumour growth by promoting cell migration and proliferation and inhibiting apoptosis and cell cycle arrest. KRT17 also activates the Akt/mTOR/hypoxia-inducible factor 1α (HIF1α) pathway by promoting proliferation and the Warburg effect, thereby promoting the growth of osteosarcoma cells (Yan et al., 2020). KRT17 can promote the proliferation and migration of esophageal cancer cells by inducing epithelial–mesenchymal transition (EMT) and activating the Akt pathway (Liu et al., 2020). It can also increase the resistance to chemotherapy drugs (Li et al., 2019). Moreover, cancer-related immunology is complex and poorly understood, partly reflected in the diversity of immune responses and the spatial and temporal heterogeneity of developing tumours (Melero et al., 2014). KRT17 plays a role with CD8+T cells, Tregs and cancer-related broblasts in malignant tumors, KRT17 and immune cells are involved in the formation and development of tumours, However, The specific mechanism of action is still unbelievable and needs further study. In sarcomas and cancers, although the origin of tumors is different, the expression of KRT17 is still high in sarcomas and cancers. It is considered that the expression of KRT17 has nothing to do with the origin of tumors, but is related to the prognosis of tumors. More and more studies have shown that KRT17 plays a very important role in the occurrence, development and prognosis of a variety of malignant tumours, making it a potential promising biomarker and therapeutic target for a variety of malignant tumours. These details will be described later in this review.

## Role of Keratin 17 in a Variety of Cancers

**FIGURE 3:**
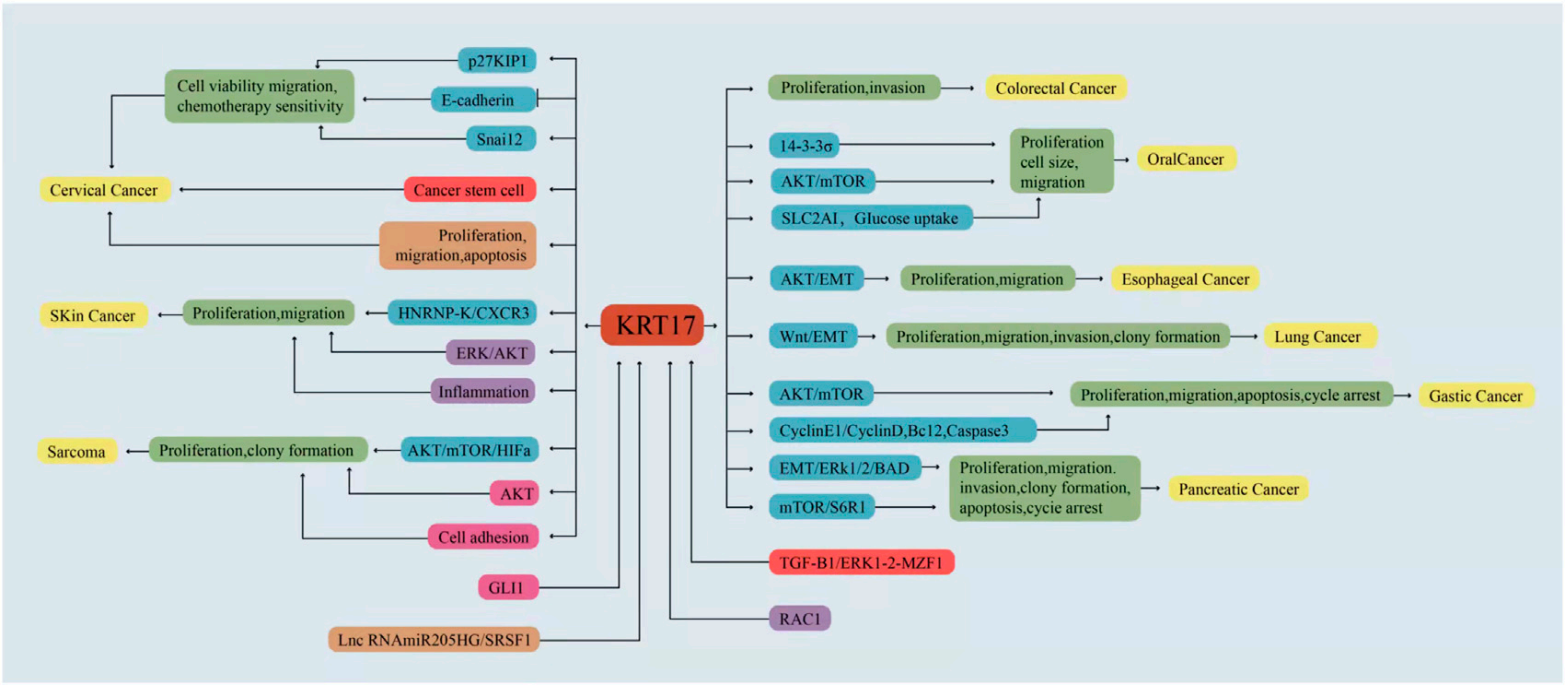
KRT17 is involved in the regulation of different signaling pathways in different malignant tumours.

A growing body of evidence has shown that KRT17 is abnormally expressed in a variety of human malignant tumours, suggesting that KRT17 is an oncogene (Liu et al., 2018; Chen et al., 2020; Chivu-Economescu et al., 2017). Genomic and proteomic studies suggest that KRT17 is highly expressed in most malignant tumor tissues or cells (Kim et al., 2013; Zhang et al., 2011; Kim et al., 2012; Takenami et al., 2019), and this high expression is related to tumour growth, metastasis and prognosis. In addition, KRT17 knockout or silencing can inhibit the growth of various tumour cell lines *in vitro* and *in vivo* (Chivu-Economescu et al., 2017; Yan et al., 2020). This section will discuss the expression and clinical significance of KRT17 in human malignant tumours, as well as the role and regulatory mechanism of KRT17 in malignant tumours (Table 1, Table 2 and Figure 3).

**TABLE 1:** Clinical significance of Keratin17 in various types of tumours.

	Clinicopathological features	References
Oral cancer	Differentiation, prognosis	Kitamura et al. (2017)
		Kitamura et al. (2012)
Oropharyngeal cancer	Prognosis	Regenbogen et al. (2018)
Laryngeal cancer	Surgical margin, prognosis	Cohen-Kerem et al. (2004)
Thyroid cancer	Lymph node metastasis, advanced N stage	Kim et al. (2015)
Esophageal cancer	Age, tumor location, smoking, lymph node metastasis, T stage, N stage and TNM stage, prognosis	Liu et al. (2020)
		Haye et al. (2020)
Lung cancer	Differentiation, gender, age, lymph node metastasis, TNM stage, prognosis	Liu et al. (2018)
		Wang et al. (2019)
Breast cancer	Triple negative state, prognosis	Merkin et al. (2017)
Gastric cancer	Tumor size, invasion depth, lymph node metastasis, clinical stage, vascular invasion, prognosis	Hu et al. (2018)
		Ide et al. (2012)
Colorectal cancer	Prognosis	Ujiie et al. (2020)
		Kim et al. (2012)
Gallbladder cancer	Differentiation, high pT stage, distant metastasis, prognosis	Kim et al. (2017)
Pancreatic cancer	Pathological grade, prognosis	Chen et al. (2020)
		Zeng et al. (2020)
		Roa-Peña et al. (2019)
		Li et al. (2020)
Renal Carcinoma	Tumor size, T stage, classification, prognosis	Sarlos et al. (2019)
Urothelial carcinoma	Diagnosis	Babu et al. (2019)
Cervical cancer	Prognosis	Escobar-Hoyos et al. (2014)
		Chaloob et al. (2016)
		Ikeda et al. (2008)
Endometrial carcinoma	Stage, prognosis	Bai et al. (2019)
Ovarian cancer	Stage, prognosis	Wang et al. (2013)
Skin cancer	Differentiation	Lan et al. (2014)

**TABLE 2:** Functional characterization of Keratin 17 in various tumours.

Malignant tumor type	Expression	Function	Mechanism	Role	References
Oral cancer	Upregulation	Proliferation migration, cell size	14-3-3σ,SLC2A1,Akt/mTOR	Oncogene	Mikami et al. (2015)
					Khanom et al. (2016)
					Mikami et al. (2017)
Esophageal cancer	Upregulation	Proliferation migration clony	Akt/EMT	Oncogene	Liu et al. (2020)
Lung cancer	Upregulation	Proliferation invasion	Wnt/EMT	Oncogene	Liu et al. (2018)
					Wang et al. (2019)
Gastric cancer	Upregulation	Proliferation migration apoptosis cell cycle arrest	Akt/mTOR,Bcl2/caspase3 cyclinE1/cyclin D	Oncogene	Chivu-Economescu et al. (2017)
					Hu et al. (2018)
Colorectal cancer	Upregulation	Proliferation invasion	N/A	Oncogene	Ujiie et al. (2020)
Pancreatic cancer	Upregulation	Proliferation migration invasion	ERK1/2/BAD,mTOR/S6K1	Oncogene	Zeng et al. (2020)
					Chen et al. (2020)
					Zhu et al. (2020)
					Li et al. (2020)
Cervical cancer	Upregulation	Proliferation apoptosis migration chemoresistance	p27^KIP1^, LncRNA miR205HG/SRSF1/KRT17,TGF- β1-ERK1/2-MZF1	Oncogene	Li et al. (2019)
					Escobar-Hoyos et al. (2015)
					Dong et al. (2019)
					Wu et al. (2017)
Sarcomas	Upregulation	Proliferation colony glycolysis cell cycle arrest	Akt/mTOR/HIF1α	Oncogene	Yan et al. (2020)
					Sankar et al. (2013)
Skin cancer	Upregulation	Proliferation invasion inflammation	HNRNP-K/CXCR3,RAC1/Erk1/2/Akt	Oncogene	Chung et al. (2015)
					Chen et al. (2014)

SLC2A1, solute carrier family 2 member 1; EMT, epithelial-mesenchymal transition; N/A, The corresponding mechanisms and targets are not yet clear; LncRNA miR205HG: long non-coding RNA MIR205 host gene; SRSF1, serine/arginine-rich splicing factor 1; TGF- β1, transforming growth factor beta-1; ERK1/2, extracellular-regulated protein kinases 1/2; MZF1, myeloid zinc finger-1; HIF1α, hypoxia-inducible factor 1α; HNRNP-K, heterogeneous nuclear ribonucleoprotein K; RAC1, ras-related C3 botulinum toxin substrate 1

### Oral Cancer

Oral squamous cell carcinoma is one of the most common malignant tumours of the head and neck (Kademani, 2007), and it has a low 5-years survival rate (Yete and Saranath, 2020). Therefore, it is of great significance to understand the biological mechanism, prognostic markers and therapeutic targets of oral cancer. The expression of KRT17 in oral cancer tissues was higher than that in paracancer tissues or normal tissues, while it was moderate, low or negative in paracancer tissues or normal tissues (Mikami et al., 2011; Wei et al., 2009; Kitamura et al., 2017; Kitamura et al., 2012). The high expression of KRT17 in oral cancer tissues is related to tissue differentiation, and KRT17 was highly expressed in low and medium differentiated tissues (Kitamura et al., 2012). In addition, the high expression of KRT17 is associated with significantly poor prognosis as shown by both univariate and multivariate analyses. Patients were followed up for a maximum of 50 months (range, 14–50 months; median, 38.6 months), The disease-free survival rate in the low expression of KRT17 group was significantly higher than that in the high expression of KRT17 group (DFSLow vs High:85 vs 34%). However, there was no significant difference in overall survival between the two groups (OSLow vs High:82 vs 68%), which may indicate that recurrence and metastasis are often successfully treated postoperatively (Kitamura et al., 2017). Another study showed that KRT17 was associated with histological grade and tumour invasion, but not with lymph node metastasis and tumour size (Mikami et al., 2017). In vitro and *in vivo* experiments, KRT17 has been shown to be highly expressed in oral cancer cell lines; KRT17 knockout can inhibit the proliferation of oral cancer cells and influence cell size. The latter effect may be modulated by 14-3-3σ(Mikami et al., 2015). Moreover, Khanom et al. (Khanom et al., 2016) selected KRT17 under-expressing Ca9-22 cells and KRT17 over-expressing HSC3 cells to establish KRT17 over-expressing Ca9-22 and KRT17 knockout HSC3 cells. Studies have shown that over-expression of KRT17 promotes cell proliferation and migration by stimulating the Akt/mTOR pathway. Over-expression of KRT17 also up-regulated the expression of SLC2A1 (solute carrier family 2 members 1) and glucose uptake. KRT17 knockout had an opposite effect in HSC3 cells. The results showed that KRT17 promoted tumour growth through the Akt/mTOR pathway and glucose uptake pathway. In OSCC, KRT17 is the downstream expressed gene of the glial-associated oncogene homologue 1 and 2 (GLI-1and GLI-2), Moreover, GLI-1 and GLI-2 can effect tumour growth by regulating KRT17 expression (Mikami et al., 2017). Hence, studies suggest that KRT17 plays a role in the occurrence and development of oral cancer and may serve as a prognostic marker and therapeutic target (Khanom et al., 2016; Snijders et al., 2009; Yan et al., 2011). In future studies, we should investigate how KRT17 affects tumour growth or progression through the role of glucose uptake.

### Oropharyngeal Cancer

Worldwide, the incidence of oropharyngeal cancer is nearly 140,000 cases/year, and the incidence of oropharyngeal cancer is on the rise in many countries (Siegel et al., 2013; Chaturvedi et al., 2013). Therefore, the discovery of effective biomarkers for the diagnosis, treatment and prognosis of oropharyngeal cancer would be helpful. Compared with normal oropharyngeal tissues, the expression of KRT17 protein was higher in oropharyngeal tumour tissues. Furthermore, patients with high KRT17 protein expression levels had poor prognosis, while patients with low KRT17 protein expression levels had a better prognosis. In addition, the expression of KRT17 protein in metastatic lymph nodes was consistent with that in the primary tumour when KRT17 protein was detected. This suggests that the expression level of KRT17 protein in primary and metastatic lesions may be related to clinical prognosis, and this may be helpful to guide clinical treatment. Moreover, the effect of human papillomavirus (HPV) on KRT17 was minimal when combining analysis of tumours with high KRT17 expression and an HPV-negative status. The identification of KRT17 as a prognostic biomarker for oropharyngeal squamous cell carcinoma (independent of HPV status) has potential clinical significance (Regenbogen et al., 2018). In conclusion, the discovery that the high expression of KRT17 can be a potential prognostic biomarker of low survival rate in patients with oropharyngeal squamous cell carcinoma may help in making treatment decisions in patients with oropharyngeal squamous cell carcinoma and improve patient prognosis, with potential clinical significance. Unfortunately, the molecular mechanisms of KRT17 in oropharyngeal cancer remain unclear to us and require further investigation.

### Laryngeal Cancer

Laryngeal carcinoma is a common malignant tumour of the head and neck, while laryngeal squamous cell carcinoma [LSCC] is a common pathological type (Almadori et al., 2005). In recent years, the incidence of LSCC, which poses a threat to human health and life, has become increasingly high (Yang et al., 2018). Therefore, it is very important to identify a molecular marker for the diagnosis, treatment and prognosis of LSCC. Cohen-Kerem et al. (Cohen-Kerem et al., 2004) found that the expression of KRT17 was higher in laryngeal cancer tissues than in normal laryngeal tissues, and the expression of KRT17 in laryngeal tissue that is proximal to the surgical margin was also higher than that in the distal laryngeal tissues after surgical resection. The results suggest that KRT17 expression level is highly sensitive and specific in the detection of laryngeal cancer and paracancerous tissues and can be used as a valuable biomarker for LSCC. In addition, the expression of KRT17 in the proximal end is higher than that in the distal end, which is important in having a certain guiding value for surgical margins. Prolonging tumour resection is an effective measure to prevent local recurrence and may hence improve the disease-free survival rate and overall survival rate of patients. However, the exact mechanism of KRT17 in laryngeal cancer is still unclear. Therefore, we still need to strengthen the study of KRT17 in laryngeal cancer mechanism and clarify the pathogenic role of KRT17 in laryngeal cancer.

### Thyroid Cancer

Papillary thyroid cancer is the most common type of malignant thyroid cancer and has a good prognosis (Hundahl et al., 2000). However, advanced age, large tumours, tumour extravasal invasion or regional lymph node metastasis, are all considered adverse prognostic factors (Ito et al., 2007; Mercante et al., 2009; Moreno-Egea et al., 1995). Therefore, it is very important to identify novel and effective molecular markers for the treatment and prognosis of papillary thyroid carcinoma. Kim et al. analyzed papillary thyroid tissue samples from 108 patients with thyroid cancer, 16 patients with nodular goitre and 81 healthy controls, and found that 65 of the 108 patients with papillary thyroid carcinoma were positive for KRT17, while KRT17 expression was negative in both nodular goitre and healthy controls, with statistically significant differences. Further studies showed that positive KRT17 expression was associated with lymph node metastasis and higher pN staging. These results suggest that KRT17 plays a crucial role in the occurrence and metastasis of papillary thyroid carcinoma (Kim et al., 2015) and that it may be a new biomarker and potential therapeutic target.

### Esophageal Cancer

Globally, the morbidity and cancer-related mortality of esophageal cancer rank seventh and sixth among all cancers, respectively. This poses a serious threat to human health (Bray et al., 2018), and the most common type is esophageal squamous cell carcinoma (ESCC) (Arnold et al., 2015). Therefore, we need to understand the molecular mechanisms of the occurrence and progression of ESCC. The expression of KRT17 in ESCC was higher than that in normal tissues, both at mRNA and protein levels. Moreover, the high expression of KRT17 has been found to be significantly correlated with clinicopathological parameters, including stage, invasion range and lymph node metastasis and is a predictor of poor prognosis in patients with advanced disease (Haye et al., 2020). Another study also confirmed that KRT17 was up-regulated in ESCC tissues, which was correlated with age, tumour location, smoking, lymph node metastasis, T stage, N stage and TNM stage, but not with differentiation or vascular and nerve invasion. The high expression of KRT17 was also associated with the poor prognosis. Survival analysis showed that ESCC patients with the high expression of KRT17 had significantly shorter survival than those with the low expression of KRT17, suggesting that KRT17 may be involved in the aggressive progression of ESCC. *In vitro* and *in vivo* experiments, the over-expression of KRT17 has been found to enhance the proliferation and migration of ESCC cells and promote tumour metastasis by activating medium-Akt signaling and inducing EMT. However, knocking out KRT17 has the opposite effect. These results suggest that KRT17 may affect tumour growth through Akt/EMT signaling pathway and a biomarker for the diagnosis and prognosis of ESCC, which provides evidence for guiding the treatment of esophageal cancer patients (Liu et al., 2020). Although KRT17 promotes the metastasis of esophageal cancer through the Akt/EMT signaling pathway, this mechanism may not be the only signaling pathway, and there may be other mechanisms, which still need to be further studied. In addition, for esophageal adenocarcinoma, whether the mechanism of KRT17 is consistent with that of ESCC also needs to be explored.

### Lung Cancer

Lung cancer is one of the most common cancers in the world, accounting for 11.6% of new cancer cases and 19.8% of cancer-related deaths each year (Fitzmaurice et al., 2017). Most lung cancer cases are diagnosed at an advanced stages, resulting in poor prognosis and poor clinical outcomes and imposing a severe burden on public health and economies worldwide (Saab et al., 2020). Therefore, it is of great significance to find new molecular markers and prognostic factors and to understand their molecular mechanisms for the diagnosis, treatment and prognosis of lung cancer. KRT17 is highly expressed in lung cancer tissues, and the high expression of KRT17 is associated with the histological type (*p* < 0.001), degree of differentiation (*p* = 0.011), sex (*p* < 0.001), age (*p* = 0.024), and lymph node metastasis (*p* = 0.04). Subgroup analysis showed that the high expression of KRT17 was only associated with sex (*p* = 0.002) in lung squamous cell carcinoma, while the high expression of KRT17 was associated with degree of differentiation (*p* = 0.036), advanced TNM stage (*p* = 0.005), and lymph node metastasis (*p* = 0.011) in lung adenocarcinoma (Wang et al., 2019). The high expression of KRT17 suggests a poor prognosis in non-small cell lung cancer, especially lung adenocarcinoma. Studies in other groups have also shown that the RNA and protein levels of KRT17 are highly expressed in lung adenocarcinoma tissues and that the high expression of KRT17 is associated with advanced TNM stage and overall survival (Liu et al., 2018). These results suggest that KRT17 can be used as a biomarker and prognostic marker for lung cancer, making it a potential new target for drug development of lung cancer, especially lung adenocarcinoma. Similarly, KRT17 is highly expressed in lung adenocarcinoma cell lines, and KRT17 knockout can inhibit cell proliferation and invasion. However, the over-expression of KRT17 can promote the proliferation and invasion of lung adenocarcinoma cells (Liu et al., 2018). Wang et al. have also shown that KRT17 knockout can inhibit the proliferation, invasion and colony formation of lung cancer cells by inhibiting the Wnt signaling pathway and EMT process. However, the overexpression of KRT17 in lung cancer cells showed the opposite result (Wang et al., 2019). Therefore, KRT17 may be a potential therapeutic target and prognostic marker for lung cancer. However, KRT17 has different characteristics in different pathological types of lung cancer, and we need to further study the expression and mechanism of KRT17 in different pathological types, so as to provide a strong basis for the treatment and prognosis of lung cancer.

### Breast Cancer

In recent years, the incidence of breast cancer has increased rapidly (DeSantis et al., 2018; Chen et al., 2016; Feng et al., 2019); however, the mortality of breast cancer patients can be reduced through early diagnosis and treatment (Robson et al., 2017; Tuttle et al., 2007). Therefore, it is very important to find new molecular markers for the diagnosis and treatment of breast cancer. Merkin et al. studied 164 cases of breast cancer patients, including 149 cases of invasive ductal carcinoma and 15 cases of invasive lobular carcinoma [three patients could not be sure of whom the triple-negative state, negative for the estrogen receptor (ER)/progesterone receptor (PR)/human epidermal growth factor receptor-2 (HER2)]. The positive expression rate of KRT17 was 82% (28/34) in the triple-negative breast cancer cells, The positive expression rate of KRT17 was 46% (52/112) in non-triple-negative breast cancer cells and 0 (0/15) in lobular carcinoma. The study showed that KRT17 was highly expressed in triple-negative breast cancer cells and that the high expression of KRT17 was associated with reduced 5-years disease-free survival in patients with advanced cancer. In addition, the expression level of KRT17 was not related to previous neoadjuvant chemotherapy. These findings suggest that KRT17 expression is associated with the triple-negative status and poor survival and is a potential prognostic biomarker for breast cancer (Merkin et al., 2017). Although KRT17 has been confirmed to be associated with breast cancer in clinical studies, it has not been confirmed in cell and animal experiments so far, and its mechanism of action in breast cancer cells needs to be further confirmed.

### Gastric Cancer

Gastric cancer (GC) is a disease that can be caused by many factors (Yusefi et al., 2018). It is the fourth most common malignancy and the third leading cause of cancer-related deaths all over the world (Fitzmaurice et al., 2015). As the disease has no obvious specific symptoms at the early stage, the diagnosis is mostly late and the prognosis is poor (Wei et al., 2020). Therefore, there is an urgent need to understand the pathogenesis of GC and find valuable therapeutic targets and prognostic markers. Compared with normal gastric tissue, the expression of KRT17 is higher in GC tissue, and the high expression of KRT17 is significantly positively correlated with tumour size, invasion depth, lymph node metastasis, clinical stage and vascular invasion (Hu et al., 2018). Munenori et al. also confirmed that the high expression of KRT17 is associated with lymph node metastasis and clinical staging and correlated with 14-three to three σ and CD10. Patients with the high expression levels of KRT17 have a decreased overall survival rate. Univariate and multivariate analyses showed that the high expression of KRT17 is a factor leading to the poor prognosis in patients with GC. Univariate analysis found that decreased overall survival was significantly associated with depth of invasion, regional lymph node metastasis, lymphatic invasion, vascular invasion, and KRT17 expression. Multivariate analysis showed that invasion beyond gastric serosa, vascular infiltration and positive expression of KRT17 were independent prognostic factors (Ide et al., 2012). Cell experiments suggest that KRT17 knockout could inhibit the proliferation and migration of GC cells, which may be achieved through the Akt/mTOR signalling pathway, and animal experiments also confirmed that KRT17 knockout could inhibit tumour growth (Chivu-Economescu et al., 2017). In addition, silencing KRT17 can alter the expression of the Bcl2 family proteins and increase the cleavage of caspase3 to induce cell apoptosis. Cell cycle arrest in the G1/S phase can also be caused by decreasing the expression of cycling E1 and cycling D (Hu et al., 2018). These studies suggest that KRT17 may play different roles in different stages of GC. Before determining KRT17 as a biomarker for progression and prognosis of GC, further studies are needed to understand the role of KRT17 in the occurrence, development and prognosis of GC.

### Colorectal Cancer

Colorectal cancer (CRC) is one of the major causes of cancer-related morbidity and mortality in the world (Brenner et al., 2014). Metastatic colorectal cancer (CRC) is the leading cause of cancer-related deaths from colorectal cancer (Emre et al., 2018; Lamami et al., 2018). Therefore, early diagnosis and early treatment of colorectal cancer are particularly important. In colorectal cancer, KRT17 is highly expressed in cancer tissues, and this high expression is negatively correlated with disease-free survival. Cox regression analysis showed that KRT17 expression had no significant correlation with age, sex, tumor location and tissue differentiation. Through univariate COX analysis, the levels expression of KRT17 was significantly associated with disease-free survival. Multivariate analysis, the expression of KRT17 was an independent factor affecting prognosis (Ujiie et al., 2020). Studies have also shown that KRT17 is negatively expressed in normal colorectal mucous and highly expressed in colorectal cancer. With the increase of T stage, the staining intensity of KRT17 also increases. However, these results did not reach statistical significance (Kim et al., 2012). In addition, KRT17 is highly expressed in colorectal cancer cells. KRT17 knockout decreases both protein and mRNA in colorectal cancer cells and inhibits the proliferation and invasion of colorectal cancer cells (Ujiie et al., 2020). These results suggest that KRT17 may be an important biomarker for colorectal cancer and a valuable therapeutic target for colorectal cancer, However, the exact mechanism of KRT17 in colorectal cancer still needs further study.

### Gallbladder Cancer

Gallbladder cancer (GBC) is the sixth most common gastrointestinal malignancy and the most common bile duct cancer in the world, with a 5-years survival rate of 5–10% in most cases (Hundal and Shaffer, 2014; Asumova et al., 2017; Hueman et al., 2009). Therefore, early diagnosis and treatment are crucial to improve the survival rate of gallbladder cancer patients. In gallbladder cancer, KRT17 expression was correlated with clinicopathological parametersl. Kim et al. found that KRT17 expression was positive in 41 of 77 tumor tissues, while negative in all 8 normal gallbladder mucosa tissues. Analysis showed that patients with positive expression of KRT17 had lower disease-specific survival. In addition, univariate analysis showed that high pT stage, KRT17 expression, poor tumor differentiation, perineural invasion and lymphatic vascular invasion were poor prognostic factors, while multivariate analysis suggested that KRT17 expression was also an important prognostic factor. Further studies found that positive KRT17 expression was associated with poor tumor differentiation, higher pT staging, more frequent distant metastasis, and a lower incidence of adenomas in the background. These results suggest that KRT17 can be used as a biomarker for poor prognosis of gallbladder adenocarcinoma (Kim et al., 2017).

### Pancreatic Cancer

Pancreatic cancer is a highly fatal disease. The 5-years survival rate is only 9%, and the global incidence has increased significantly over the past few decades (Chen et al., 2016; Siegel et al., 2019). Surgical treatment is the main form of treatment for pancreatic cancer, but less than 20% of patients receive surgery because pancreatic cancer is often insidious in its early stages and usually advanced at the time of diagnosis (Kamisawa et al., 2016; Gillen et al., 2010). Many pancreatic cancer patients still have a recurrence after surgical resection (Siegel et al., 2014) and pancreatic cancer is not sensitive to radiotherapy and chemotherapy (Zhu et al., 2020). Therefore, understanding the mechanism of pancreatic cancer occurrence, development and progression may be of great help to its treatment and prognosis. Both KRT17 protein and mRNA were highly expressed in pancreatic cancer tissues, while they were expressed at low levels in normal pancreatic tissues. The high expression of KRT17 was significantly correlated with pathological grade, but not with tumour size, TNM stage, clinical stage, lymph node metastasis or distant metastasis. In addition, univariate analysis showed that both pathological grade and up-regulated KRT17 expression were associated with overall survival. Multivariate analysis showed that pathological grade, lymph node metastasis, and up-regulated KRT17 expression was independent prognostic factors for overall survival (Zeng et al., 2020; Chen et al., 2020; Yang et al., 2012). In addition, the high expression of KRT17 is associated with basal-like molecular sub-types of pancreatic cancer, while the low expression of KRT17 is typically associated with pancreatic cancer. This study proved that KRT17 and molecular typing can accurately reflect the prognosis of pancreatic cancer, but it is independent of tumour grade, stage and marginal state (Roa-Peña et al., 2019). Similarly, KRT17 mRNA and protein levels were highly expressed in pancreatic cancer cell lines. KRT17 knockout can inhibit cell migration, proliferation and colony formation, induce cell apoptosis and cell cycle arrest, and inhibit tumour growth in xenograft mice. In addition, KRT17 knockout can induce EMT and affect ERK1/2/BAD signaling pathway (Zeng et al., 2020; Chen et al., 2020). Furthermore, KRT17 knockout can also inhibit tumour cell proliferation, migration and invasion through the mTOR/S6K1 signaling pathway (Zhu et al., 2020; Li et al., 2020). These findings suggest that KRT17 may serve as a prognostic marker and therapeutic target for pancreatic cancer.

### Renal Carcinoma

Kidney cancer makes up a small percentage of all cancer types; only 2.2% (Bhatt and Finelli, 2014). Renal cell carcinoma (RCC) is also the most lethal of all urinary malignancies. Approximately, 30% of patients develop distant metastases, and half of these patients die as a result (Bray et al., 2018; Bhatt and Finelli, 2014). Therefore, it is very important to identify new biomarkers and prognostic markers for renal cancer, this will help to understand the occurrence, development and prognosis of renal cancer. KRT17 is positively expressed in papillary RCC, and the continuous expression of KRT17 from the foetal to adult stages in papillary RCC confirms the association between foetal renal development and tumorigenesis of papillary RCC tumorigenesis. In addition, in traditional RCC with positive expression of KRT17, the 5-years and 10-years survival rates decreased. Multivariate analysis showed that the positive expression of KRT17 in invasive conventional RCC was significantly correlated with postoperative tumour recurrence. The above findings suggest that the multiple functions of KRT17 in renal carcinoma clearly describe the different natural history between papillary and conventional RCC (Sarlos et al., 2019). These results suggest that KRT17 may play an important role in the occurrence, progression and prognosis of RCC.

### Urothelial Carcinoma

Urothelial carcinoma (UC) is a common urinary tract tumor (Siegel et al., 2019), and about a quarter of patients have a history of muscular-invasive bladder cancer or metastatic bladder cancer (Antoni et al., 2017). Although treatment methods have improved, the prognosis remains poor, especially in patients with advanced or metastatic UC, with a median overall survival (OS) of about 15 months (Burger et al., 2013). Therefore, there is an urgent need for new and effective early diagnostic markers to help in finding strong candidates for the treatment of UC. Compared with normal urothelial tissues, KRT17 expression was up-regulated in non-papillary invasive UC, the high-grade papillary urothelial carcinoma (PUC-LG), the low-grade papillary urothelial carcinoma (PUC-HG) and papillary urothelial tumour with low malignant potential (PunLMP) tissues. Moreover, KRT17 expression was higher in non-papillary invasive UC than in the high-grade and the low-grade papillary UC. Immunohistochemistry of KRT17 in tissues can distinguish UC (PUC-LG, PUC-HG and UC) from normal urothelial mucous and is highly sensitive and specific, but positive test results cannot rule out the diagnosis of PunLMP. Interestingly, KRT17 urine immunocytochemistry had a sensitivity of 100%, specificity of 96%, the positive predictive value of 91% and the negative predictive value of 100% for UC (Babu et al., 2019). KRT17 may be used as a sensitive and specific biomarker for UC, especially for the detection of urine of UC patients, with convenient sampling, faster detection and broader clinical application prospects.

### Cervical Cancer

Cervical cancer ranks fourth in the incidence of malignancies and cancer-related deaths among women (Bray et al., 2018), accounting for 4% of all cancers diagnosed worldwide (Bermudez et al., 2018). Although the disease can be prevented through screening and treatment of precancerous lesions, cervical cancer remains the most common cause of cancer death in women (Aggarwal, 2014). Therefore, it is very important to further study the molecular mechanism of cervical cancer. The expression of KRT17 was up-regulated in the high-grade squamous intraepithelial lesion and squamous cell carcinoma (SCC) compared to the normal cervical epithelium and low-grade squamous intraepithelial lesion. In cervical SCC, Cox proportional hazards model analysis shows that the expression of KRT17 in SCC was significantly associated with OS of patients. Patients with the low expression of KRT17 had an estimated 5-year survival rate of 96.97%, while patients with the high expression of KRT17 had an estimated 5-year survival rate of 64.31%. The 10-years survival rate was estimated to be 96.97% for patients with the low expression of KRT17 and 52.61% for patients with the high expression of KRT17, but there was no correlation with the tumour stage, histological grade or lymph node metastasis (Escobar-Hoyos et al., 2014; Chaloob et al., 2016; Ikeda et al., 2008). It is suggested that the high expression of KRT17 is invasive, and the treatment of the high expression of KRT17 in cervical cancer patients should be cautious in clinical practice. In cervical cancer cell lines, KRT17 knockout decreased the viability and migration ability of cervical cancer cells and down-regulated Snail2 and up-regulated E-cadherin. Moreover, KRT17 knockout has been found to inhibit cell viability and migration of cervical cancer cells and increase paclitaxel sensitivity to chemotherapy (Li et al., 2019). KRT17 knockout can also inhibit cell proliferation and increase sensitivity to cisplatin chemotherapy, which is achieved by KRT17 regulating the subcellular localisation and degradation of p27KIP1 (Escobar-Hoyos et al., 2015). In addition, studies have shown that KRT17 also effects the proliferation, apoptosis and migration of cervical cancer cells through a regulatory axis involving lncRNA miR205HG -SRSF1-KRT17 and TGF- B1-ERK1/2-MZF1 -KRT17, thus enhancing the characteristics of cervical cancer stem cells (Dong et al., 2019; Wu et al., 2017). These results suggest that KRT17 plays an important role in the pathogenesis and progression of cervical cancer, and may be a potential diagnostic marker or a valuable therapeutic target.

### Endometrial Carcinoma

Endometrial carcinoma (EC) is a common malignancy in women (Dörk et al., 2020). The main pathological types include endometrioid adenocarcinoma and serous carcinoma. Serous carcinoma is poorly differentiated and, therefore, has a poor prognosis (Setiawan et al., 2013; Black et al., 2016). Still, 15%–25% of patients are diagnosed with advanced disease (stage III or IV), with a 5-years survival rate of 40–79% for FIGO III patients and 0–24% for FIGO IV patients (Van et al., 2006). Besides, the higher the histological grade, the worse the patient’s prognosis. Therefore, early diagnosis and treatment are very important to improve endometrial cancer survival rates. Compared with endometrial stroma, myometrium and uterine sarcoma, KRT17 was positively expressed in EC. In patients with high-grade endometrial cancer, high expression levels of KRT17 mRNA was associated with decreased OS, with a median survival of 4.2 years for high expression of KRT17 mRNA and 9.2 years for low expression of KRT17 mRNA. Unfortunately, both univariate and multivariate analyses showed that KRT17 mRNA expression was not significantly associated with survival. Immunohistochemical results indicated that high expression of KRT17 protein was also associated with decreased OS, with a median survival time of 1.7 years for high expression and 4.6 years for low expression. In univariate and multivariate analyses, KRT17 protein expression status was independently associated with survival and cancer stage. Moreover, in multivariate analysis, the hazard ratios of KRT17 protein expression were higher than the hazard ratios of KRT17 mRNA expression, suggesting that KRT17 protein, rather than KRT17 mRNA, expression is an independent prognostic factor (Bai et al., 2019). Studies have shown that KRT17 protein is an independent prognostic biomarker in patients with high-grade endometrial cancer.

### Ovarian Cancer

Ovarian cancer is a common gynecological cancer and even the main cause of death of gynecological cancers. Although the prognosis is good at early diagnosis, the vast majority of patients are diagnosed with advanced stage, with a 5-years survival rate of about 30–40% (Nimmagadda and Penet, 2020). Therefore, there is urgent to find molecular markers for the early diagnosis of ovarian cancer in order to improve the survival rate of ovarian cancer patients. Both mRNA and protein expression levels of KRT17 were significantly higher in ovarian cancer tissues than in non-cancerous tissues. In addition, the high expression of KRT17 was associated with clinicopathological parameters, such as stage and prognosis; the later the clinical stage of the high expression of KRT17, the worse the prognosis. Multivariate analysis showed that the high expression level of KRT17 was an independent prognostic factor for OS in ovarian cancer patients. The results confirm that the high expression of KRT17 is significantly associated with tumour progression and poor prognosis in ovarian cancer (Wang et al., 2013). The above results show that KRT17 may be a molecular marker for the occurrence, development and prognosis of ovarian cancer, which provides useful information for clinical applications.

### Sarcomas

Sarcoma is a mesenchymal malignancy with high heterogeneity. Although it accounts for only 1% of all malignancies, it is the second most common malignancy in children and adolescents (Grünewald et al., 2020). When the lesion is localized, the prognosis of surgical resection plus adjuvant radiotherapy is fair, with a 5-years survival rate of about 60% (Woll et al., 2012). However, when the disease is advanced, the prognosis is very poor, with a median survival of about 1 year (Judson et al., 2014). Therefore, there is an urgent need to further elucidate the molecular mechanisms of sarcoma in order to identify biomarkers for the early diagnosis. In osteosarcomas, KRT17 mRNA and protein expression levels are higher than in the adjacent normal bone tissue, but the expression level of KRT17 was not analysed and compared with clinicopathologic parameters in this study. The mRNA and protein expression levels of KRT17 in osteosarcoma cells were also higher than those in the normal bone hFOB cells. KRT17 knockout can inhibit proliferation, colony formation and glycolysis and can induce cell phase arrest of osteosarcoma cells. Phosphorylation (P) of Akt, *p*-mTOR, HIF1α and HIF1α glucose transporter 1 target gene expression also decreased. The re-expression of *p*-Akt, *p*-mTOR or HIF1α inhibited the inhibitory effect of KRT17 on cell proliferation and glycolysis. The results showed that KRT17 knockout inhibited the proliferation and glycolysis of osteosarcoma cells by inhibiting the Akt/mTOR/HIF1α pathway (Yan et al., 2020). In Ewing’s sarcoma, KRT17 is the downstream expression gene of the glial-associated oncogene homologue 1 (GLI-1), which positively regulates KRT17 expression. GLI-1 knockout significantly reduces Akt phosphorylation in Ewing’s sarcoma cells, and this effect can be salvaged by the overexpression of GLI-1 and KRT17. Therefore, KRT17 mediated activation of Akt signaling is necessary for regulating cell adhesion in Ewing’s sarcoma. Interestingly, the KRT17 mutant protein failed to phosphorylate Akt and retained the ability to salvage KRT17 knockout mediated transformation loss to a degree comparable to that of wild-type KRT17, suggesting that the oncogene transformation of KRT17 was independent of the Akt pathway (Sankar et al., 2013). Although KRT17 plays an important role in the occurrence and development of sarcomas, the mechanism of KRT17 in different types of sarcomas is still unclear and needs further study. In addition, we need to study KRT17 in human tumour tissues to understand the relationship between KRT17 and clinicopathological parameters.

### Skin Cancer

Skin cancer is the most common cancer in humans. The most common types are keratinocyte derived basal cell carcinoma and SCC (Rangwala and Tsai, 2011), but their molecular mechanisms remain unclear. Therefore, there is an urgent need to understand the exact molecular mechanism of skin cancer occurrence and development in order to further improve the cure rate and survival rate in patients. Generally, there is little or no expression of KRT17 in normal skin, no expression of KRT17 in the dermis and high expression of KRT17 in skin SCC, especially in poorly differentiated skin SCC cells. Therefore, keratin 17 is a marker of skin tumour cell proliferation (Lan et al., 2014). KRT17 also promotes the proliferation and invasion of skin tumours through the KRT17/HNRNP-K/CXCR3 pathway (Chung et al., 2015). In addition, RAC1 is a member of the small GTP-Rho family, and both RAC1 and KRT17 are highly expressed in cutaneous SCC. The high expression levels of RAC1 may be related to the elevated expression of KRT17. Moreover, Rac1 might exert its regulatory effects through modulating KRT17 Erk1/2 and Akt activity, hence affecting cell proliferation and differentiation. The high expression levels of Rac1 increase the levels and interactions of CD11b + Gr1 + cells, induce KRT17 to regulate inflammation and promote the formation of skin tumors. CD11b + Gr1 + cells and keratin cells activate the Wnt signalling pathways, leading to over-expression of Rac1 and KRT17 (Chen et al., 2014). These results suggest that KRT17 may be used as a biomarker for skin tumour, and that KRT17 may be more effective, especially in combination with other specific biomarkers.

## Conclusion

**FIGURE 4:**
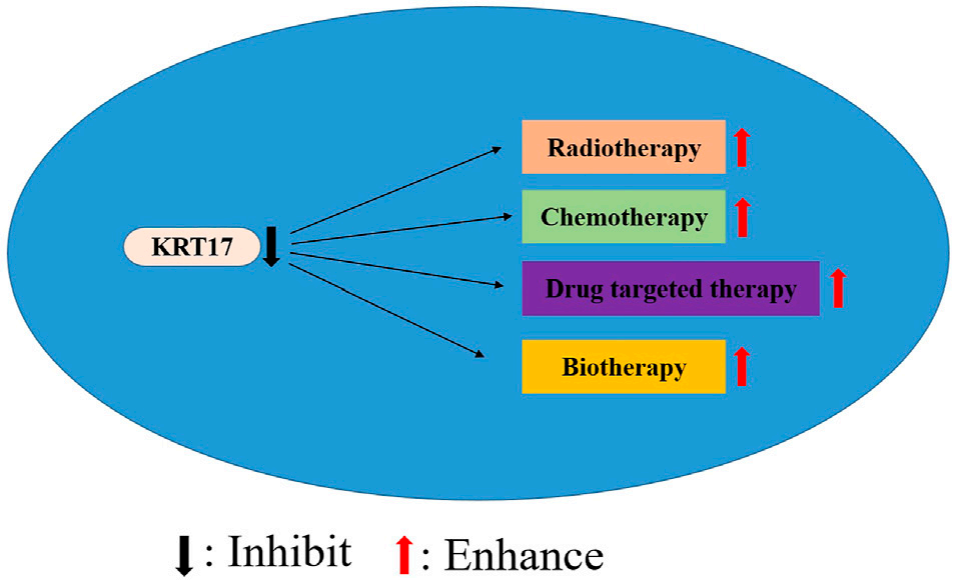
Inhibition of KRT17 expression may improve the efficacy of radiotherapy, chemotherapy, drug targeted therapy and biotherapy.

KRT17 is highly expressed in a variety of human malignancies and may be considered a valuable gene for research. However, KRT17 is underexpressed in some tumors, and it is considered that KRT17 may play a role as a tumor suppressor gene in these tumors. The high expression of KRT17 is associated with poor clinicopathological parameters of various malignant tumours, such as tumour size, depth of invasion, lymph node metastasis, tumour differentiation, distant metastasis and survival rate. Hence, KRT17 can be used as a potential prognostic biomarker for malignant tumours. However, the prognostic significance of KRT17 in malignant tumours needs to be confirmed by a large number of clinical case studies. Similarly, KRT17 also shows the high expression levels in malignant tumour cell lines. Knockout or silenced KRT17 can inhibit the proliferation, migration, adhesion and invasion of tumour cells, and promote the apoptosis of tumour cells. It may regulate a wide variety of tumour-related molecules or pathways that play a role in tumour formation. Hence, the upstream and downstream regulatory pathways of KRT17 are of significance, but further validation is needed, especially in unreported tumours or more precise mechanism studies. In addition, KRT17 knockout can increase the chemotherapeutic sensitivity for paclitaxel and cisplatin, suggesting that KRT17 is also a feasible and valuable therapeutic target, but the exact mechanism of action remains unclear and needs further elucidation. However, whether KRT17 can improve the sensitivity of radiotherapy and the efficacy of immunotherapy and biotherapy may be a new research direction of KRT17 in the future (Figure 4). In addition, KRT17 may have other pathways of action during the occurrence and development of human tumours, which still need to be studied and explored.

In conclusion, KRT17 is an important, but really understudied gene. It plays a key role in promoting the growth and metastasis of tumour cells, highlighting its role in the pathogenesis of tumour. However, KRT17 as a biomarker and therapeutic target for tumour prognosis remain a major challenge, as the exact molecular mechanisms of KRT17 action remain unclear. At present, there is no unified and standardized detection method for clinical screening of KRT17 expression.
